# Platelet activation and thrombosis in microvascular obstruction

**DOI:** 10.1093/ehjci/jeag225

**Published:** 2026-08-30

**Authors:** Craig Balmforth, Alessandro Giaj Levra, Beth Whittington, Aidan Morgan, Mark G Macaskill, Evangelos Tzolos, Alex J Jordan, Tariq Ramtoola, Tim Clark, Michelle Rooney, Christophe Lucatelli, Edwin J R van Beek, Piotr J Slomka, Stephanie L Sellers, Norman Koglin, Andrew W Stephens, Iris Hardewig, David E Newby, Michelle C Williams, Marc R Dweck

**Affiliations:** British Heart Foundation Centre of Research Excellence, University of Edinburgh, Institute for Neuroscience and Cardiovascular Research, Edinburgh, United Kingdom of Great Britain & Northern Ireland; British Heart Foundation Centre of Research Excellence, University of Edinburgh, Institute for Neuroscience and Cardiovascular Research, Edinburgh, United Kingdom of Great Britain & Northern Ireland; Department of Biomedical Sciences, Humanitas University, Milan, Italy; British Heart Foundation Centre of Research Excellence, University of Edinburgh, Institute for Neuroscience and Cardiovascular Research, Edinburgh, United Kingdom of Great Britain & Northern Ireland; British Heart Foundation Centre of Research Excellence, University of Edinburgh, Institute for Neuroscience and Cardiovascular Research, Edinburgh, United Kingdom of Great Britain & Northern Ireland; British Heart Foundation Centre of Research Excellence, University of Edinburgh, Institute for Neuroscience and Cardiovascular Research, Edinburgh, United Kingdom of Great Britain & Northern Ireland; British Heart Foundation Centre of Research Excellence, University of Edinburgh, Institute for Neuroscience and Cardiovascular Research, Edinburgh, United Kingdom of Great Britain & Northern Ireland; British Heart Foundation Centre of Research Excellence, University of Edinburgh, Institute for Neuroscience and Cardiovascular Research, Edinburgh, United Kingdom of Great Britain & Northern Ireland; British Heart Foundation Centre of Research Excellence, University of Edinburgh, Institute for Neuroscience and Cardiovascular Research, Edinburgh, United Kingdom of Great Britain & Northern Ireland; Edinburgh Imaging, Queen's Medical Research Institute, Edinburgh, United Kingdom of Great Britain & Northern Ireland; Edinburgh Imaging, Queen's Medical Research Institute, Edinburgh, United Kingdom of Great Britain & Northern Ireland; Edinburgh Imaging, Queen's Medical Research Institute, Edinburgh, United Kingdom of Great Britain & Northern Ireland; British Heart Foundation Centre of Research Excellence, University of Edinburgh, Institute for Neuroscience and Cardiovascular Research, Edinburgh, United Kingdom of Great Britain & Northern Ireland; Cedars-Sinai Medical Centre, Los Angeles, CA, USA; Division of Cardiology, Department of Medicine, University of British Columbia, Vancouver, BC, Canada; Lantheus Germany GmbH, Berlin, Germany; Lantheus Germany GmbH, Berlin, Germany; Lantheus Germany GmbH, Berlin, Germany; British Heart Foundation Centre of Research Excellence, University of Edinburgh, Institute for Neuroscience and Cardiovascular Research, Edinburgh, United Kingdom of Great Britain & Northern Ireland; British Heart Foundation Centre of Research Excellence, University of Edinburgh, Institute for Neuroscience and Cardiovascular Research, Edinburgh, United Kingdom of Great Britain & Northern Ireland; British Heart Foundation Centre of Research Excellence, University of Edinburgh, Institute for Neuroscience and Cardiovascular Research, Edinburgh, United Kingdom of Great Britain & Northern Ireland

## Abstract

**Aims:**

Microvascular obstruction (MVO) remains a major complication following coronary reperfusion, but its pathogenesis is poorly defined. We evaluated myocardial platelet activation and thrombosis using fluorine-18-labelled GP1 ([^18^F]GP1) positron emission tomography (PET) in patients with anterior ST-segment elevation myocardial infarction.

**Methods and results:**

In a single-centre observational cohort study, 100 patients with acute anterior ST-segment elevation myocardial infarction underwent hybrid cardiac [^18^F]GP1 PET and magnetic resonance (MR) imaging with gadolinium enhancement and repeat imaging at 3–6 months. Myocardial [^18^F]GP1 uptake was quantified using target-to-background ratio (TBR_max_) and percentage myocardial uptake (burden) and compared with MR measures of infarction, MVO and adverse ventricular remodelling. One hundred patients (60 ± 10 years, 91% male) underwent [^18^F]GP1 PET/MR imaging at a median of 13 days following index presentation. Increased myocardial [^18^F]GP1 uptake was observed in 44% of participants (TBR_max_ 1.89 ± 0.50). All 40 patients with MR-defined MVO demonstrated myocardial [^18^F]GP1 uptake. Myocardial [^18^F]GP1 uptake burden correlated with (ρ = 0.84; *P* < 0.001), and exceeded (by 18%), MR-defined burden of MVO. Myocardial [^18^F]GP1 uptake was associated with infarct size, lower left ventricular ejection fraction and longer time-to-reperfusion. Angiographic no-reflow was the strongest independent association of MVO (adjusted odds ratio 16.15; *P* < 0.001). Persistent myocardial [^18^F]GP1 uptake at follow up (22%, 7/32) was associated with lower left ventricular ejection fraction. Resolution of cardiac MR-defined MVO was observed in 78% of patients at follow-up and was universally concordant with resolution of myocardial [^18^F]GP1 uptake.

**Conclusion:**

MVO following ST-segment elevation myocardial infarction is universally associated with myocardial platelet activation, extending beyond structural abnormalities identified by cardiac MR. These findings are consistent with an important mechanistic contribution of platelet-mediated microvascular thrombosis to no-reflow and MVO, highlighting a potentially targetable biological pathway in a condition for which effective therapies remain elusive.

## Graphical Abstract

**Graphical Abstract:**
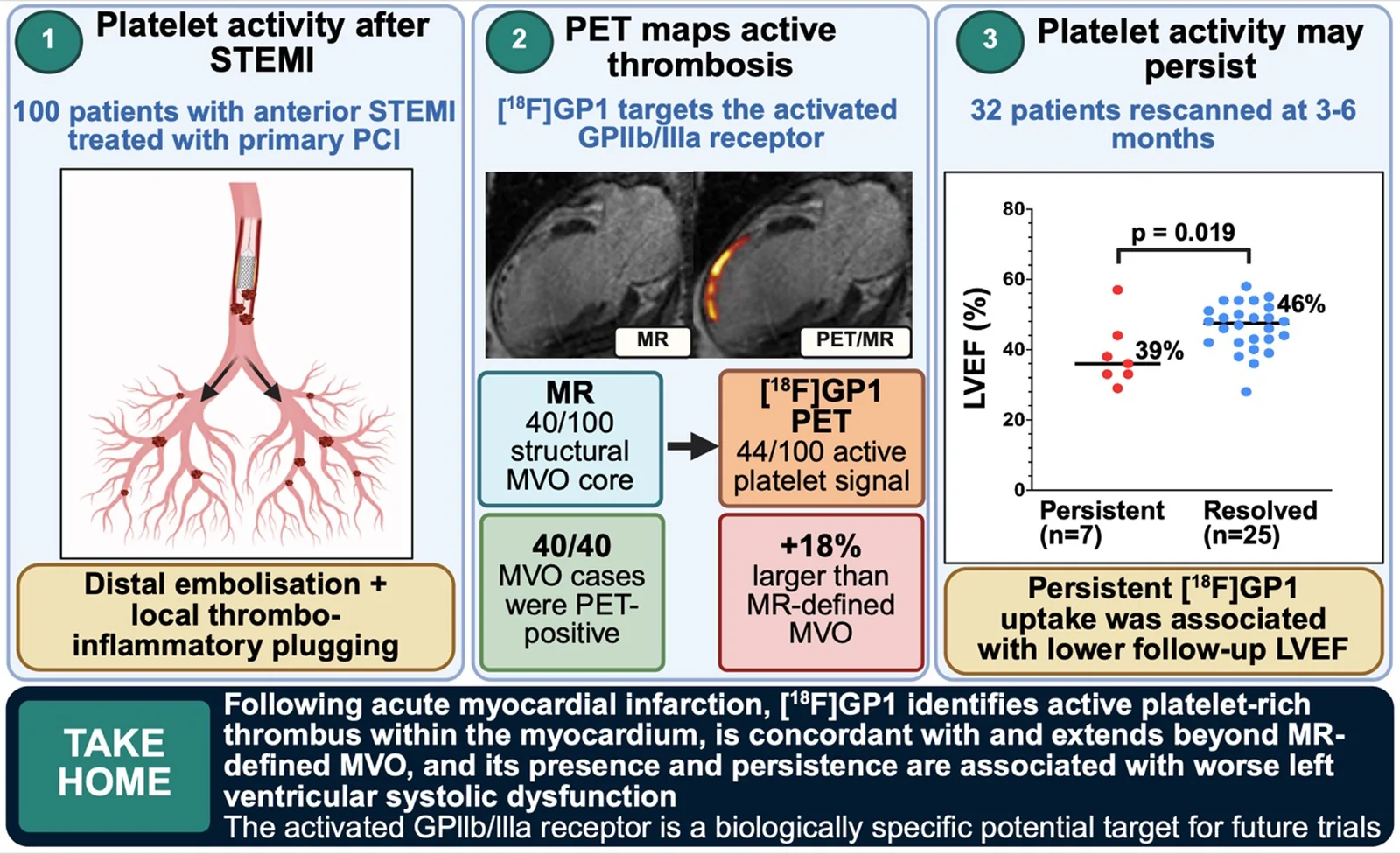
Platelet activation and thrombosis in microvascular obstruction: [^18^F]GP1 PET/MR after anterior STEMI: mechanism, spatial extent and persistence. *GPIIb/IIIa, glycoprotein IIb/IIIa; LVEF, left ventricular ejection fraction; MR, magnetic resonance; MVO, microvascular obstruction; PCI, percutaneous coronary intervention; PET, positron emission tomography; STEMI, ST-segment elevation myocardial infarction.*

**See the editorial comment for this article ‘The molecular footprint of thrombosis: what does myocardial platelet imaging after STEMI tell us?’, by H. Bulluck and D. J. Hausenloy, https://doi.org/10.1093/ehjci/jeag229.**

## Introduction

In ST-segment elevation myocardial infarction, early reperfusion by primary percutaneous coronary intervention achieves restoration of normal epicardial flow in approximately 95% of patients and lowers in-patient mortality, forming the cornerstone of guideline recommended treatment.^1–3^ However, despite restoration of coronary arterial patency, failure to restore myocardial perfusion because of coronary microvascular obstruction remains common. Microvascular obstruction affects up to 40–50% of patients and is independently associated with larger infarct size, adverse left ventricular remodelling and adverse cardiovascular outcomes, representing a persistent unmet therapeutic need in the management of acute myocardial infarction.^1,4–6^

Microvascular obstruction following ST-segment elevation myocardial infarction is a heterogeneous and multifactorial process that remains incompletely understood. It encompasses several interrelated pathophysiological mechanisms including endothelial injury, inflammatory cell infiltration, vasomotor dysfunction, myocardial oedema, intra-myocardial haemorrhage, and embolization of atherothrombotic debris.^4^ Mechanistically, obstruction of the coronary microcirculation arises from both extrinsic and intrinsic factors. Distal embolization of proximal thrombotic and atherosclerotic debris during plaque rupture and coronary intervention contributes to mechanical obstruction of the microvasculature, while ischaemia–reperfusion injury induces endothelial dysfunction characterized by calcium overload, oxidative stress, mitochondrial injury and endothelial swelling, leading to capillary narrowing and collapse.^7^ These processes are further exacerbated by myocardial oedema, microvascular spasm and impaired vasoregulation, which can ultimately lead to microvascular plugging by platelets, leukocytes, and erythrocytes.^7,8^

Activated platelets contribute to distal microembolization during coronary intervention and promote *in situ* microvascular thrombosis through aggregation and interaction with leukocytes, amplifying thrombo-inflammatory signalling within the infarct zone.^9–11^ Experimental and histopathological studies demonstrate platelet-rich microthrombi and microvascular plugging within infarcted myocardium following reperfusion, implicating platelet-mediated microvascular plugging in the pathogenesis of no-reflow. Clinical studies further support this concept, demonstrating that heightened platelet reactivity following ST-segment elevation myocardial infarction is associated with a greater extent of microvascular obstruction and infarct size despite contemporary dual antiplatelet therapy.^11^ However, direct *in vivo* human evidence linking activated platelets to myocardial microvascular obstruction remains limited as contemporary imaging techniques predominantly characterize structural injury or inflammatory activity rather than thrombotic biology.^10,12–14^

The radiotracer fluorine-18 GP1 ([^18^F]GP1) is derived from elarofiban and binds with high-affinity and specificity to activated glycoprotein IIb/IIIa receptors expressed exclusively on activated platelets.^15^ In a range of cardiovascular conditions, [^18^F]GP1 positron emission tomography (PET) can non-invasively detect acute venous and arterial thrombosis *in vivo*.^16–20^ We hypothesized that platelet-mediated microvascular thrombosis is a central mechanistic contributor to myocardial microvascular obstruction following anterior ST-segment elevation myocardial infarction. Accordingly, we aimed to evaluate *in vivo* myocardial activated platelet imaging using hybrid [^18^F]GP1 PET and magnetic resonance (MR) imaging, and to define the spatial and quantitative relationship between myocardial [^18^F]GP1 PET uptake and cardiac MR-defined markers of infarction, microvascular obstruction, and adverse remodelling. Finally, we explored the temporal evolution of myocardial platelet activity following myocardial infarction and its relationship to cardiac remodelling.

## Methods

### Study design and population

This was a single-centre prospective observational cohort study (NCT04829825). We recruited patients over the age of 40 years who presented with acute anterior ST-segment elevation myocardial infarction as defined by the fourth universal definition of myocardial infarction from the Edinburgh Heart Centre (Edinburgh, UK).^21^ All patients had undergone emergency coronary angiography and percutaneous coronary intervention. Baseline transthoracic echocardiography was performed as part of routine clinical care in all patients. Enrolled participants who had evidence of myocardial [^18^F]GP1 PET uptake on baseline clinical imaging attended for repeat [^18^F]GP1 PET/MR scan at 3–6 months. Exclusion criteria included pregnancy or breastfeeding, contraindications to magnetic resonance imaging, use of concomitant anticoagulant therapies, history of atrial fibrillation, and estimated glomerular filtration rate <30 mL/min/1.73 m^2^.

The study was conducted in accordance with the Declaration of Helsinki, the approval of the South-east Scotland Regional Ethics Committee (20/SS/0083) and the written informed consent of all participants.

### Histological correlation of [^18^F]GP1 uptake in human myocardium

*Ex vivo* assessment of activated platelets in human myocardial infarction was performed using explanted myocardial tissue from patients with acute myocardial infarction (<4 weeks; *n* = 3) as well as healthy myocardium (*n* = 2) obtained at the time of orthotopic cardiac transplantation or autopsy. Tissue was obtained from the Bruce McManus Cardiovascular Biobank (UBC centre for Heart Lung Innovation at St. Paul′s Hospital) under separate local ethical approval.

Haematoxylin and eosin staining was performed on 10-µm paraffin sections of infarcted and remote myocardium. CD41 immunohistochemistry was performed using a rabbit monoclonal [EPR4330] antibody to CD41 (Abcam, lot #GR3259786) at a dilution of 1:100. Staining was conducted using a Leica Bond Rx system using Bond Epitope Retrieval Solution 1 (pH = 6, Catalog No: AR9961) and Bond Polymer Refine Red Detection (Catalog No: DS9390). The omission of the primary antibody served as negative controls.

Autoradiography was performed on infarcted and remote myocardial specimens. Two paraffin embedded sections (5-µm thickness) were de-paraffinized and sections were incubated in phosphate-buffered saline with 1 nM [^18^F]GP1 for 60 min at room temperature with and without 10 µM unlabelled GP1 as a blocking agent. After two washes in phosphate-buffered saline and one in deionized water, the slides were dried and exposed to a high-resolution autoradiography plate (BAS-IP_SR 2040; Cytiva). The plates were imaged on an autoradiography imager (Amersham Typhoon IP Biomolecular Imager, Cytiva).

### Positron emission tomography and magnetic resonance imaging

Participants underwent hybrid [^18^F]GP1 PET/MR imaging (Biograph m-MR, Siemens Healthineers) with a 40-min bed position centred over the heart, 60 min after the administration of 250 MBq of [^18^F]GP1. The cardiac MR protocol included a free-breathing radial volumetric interpolated breath-hold examination sequence for attenuation correction, and long and short-axis stack cine imaging. The protocol also included early gadolinium enhancement images in long-axis views (two-chamber, three-chamber, and four-chamber views) acquired 2–3 min following administration of 0.1 ml/kg gadobutrol (Gadovist; Bayer Inc., Mississauga, Canada) as well as late gadolinium enhancement images acquired in long-axis views (two-chamber, three-chamber, and four-chamber views) and two full short-axis stacks in two phase-encoding directions 7–15 min post-contrast administration.

### Magnetic resonance image analysis

Cardiac image analysis was performed by expert readers (C.B., B.W., E.T., A.G.L., M.R.D.). Magnetic resonance analysis was performed independently of the positron emission analysis using cvi42 (Circle Cardiovascular Imaging, Canada). Left ventricular volumes, mass, and ejection fraction were measured using manual adjustment of automated epicardial and endocardial contours on the short-axis cine stack. The late gadolinium enhancement signal intensity threshold for infarct size quantification was set at 5 standard deviations above the reference region of interest in the remote unaffected myocardium. This threshold was used for the quantification of both late gadolinium enhancement percentage and microvascular obstruction percentage quantification.^22^ Left ventricular infarct burden was then quantified as the myocardial infarct volume divided by the left ventricular myocardial volume, multiplied by 100 to be expressed as a percentage. Areas of microvascular obstruction were identified as areas of hypoenhancement within the late gadolinium enhancement, quantified as a volume then expressed as a percentage of left ventricular myocardial volume (MR burden of microvascular obstruction).

### Positron emission tomography image analysis

PET image analyses were performed using both global and regional approaches to assess the intensity, volume, and burden of myocardial platelet activation. QPET (Cedars Sinai, LA) was used for the global analysis and FusionQuant for the regional assessments (Cedars Sinai, LA). Both platforms allow precise image co-registration, automated motion correction, and advanced cardiac PET quantification.

#### Global myocardial [^18^F]GP1 uptake

Semiautomated segmentation of the myocardial positron emission tomography signal arising from the left ventricle was performed in three orthogonal planes using an adjustable left ventricular region of interest as described previously.^23^ A mask was used to exclude all counts outside of the cardiac silhouette.

The intensity of myocardial [^18^F]GP1 uptake across the left ventricle as a whole was quantified using maximum (SUV_max_) and mean (SUV_mean_) standard uptake values.^22^ Maximum (TBR_max_) and mean (TBR_mean_) target-to-background ratios were then calculated by correcting the myocardial uptake for the background blood pool activity measured in the right atrium. The total volume of [^18^F]GP1 uptake was quantified based on pixels with [^18^F]GP1 uptake above a threshold of the right atrial blood pool SUV_mean_ + 2×standard deviations. This was used to calculate the global burden of myocardial platelet activation (percentage of the myocardium with increased [^18^F]GP1 uptake) in an approach similar to that for the infarct burden and burden of microvascular obstruction on cardiac MR (volume of increased myocardial [^18^F]GP1 uptake divided by the total myocardial volume, multiplied by 100).^24^

Finally, QPET software was used to assess [^18^F]GP1 uptake (SUV_max_, SUV_mean_, TBR_max_, TBR_mean_) within individual segments of the American Heart Association 17-segment myocardial model.

#### Regional myocardial [^18^F]GP1 uptake

Regional analysis of the quantitative [^18^F]GP1 uptake across the left ventricular myocardium was guided by MR. PET images were overlaid directly onto magnetic resonance short-axis late gadolinium enhancement sequences for all scans. First, the PET images and late enhancement sequences were fused in three orthogonal planes. Then, regions of interest were drawn using free-form polygons guided by the late gadolinium enhancement images, to measure [^18^F]GP1 uptake in the infarct zone (defined as the area of late gadolinium enhancement) and the remote myocardium which was defined as the segment of normal myocardium without late gadolinium enhancement diametrically opposite to the infarct on short-axis images according to the American Heart Association 17-segment model. SUV_max_ and TBR_max_ values were then calculated for all regions of interest.

### Statistical analysis

All statistical analyses were performed using R studio version 2023.06.1 (R Foundation for Statistical Computing, Vienna, Austria). Continuous variables are presented as mean ± standard deviation for normally distributed data or median [interquartile interval] for non-normally distributed data, and categorical variables are presented as counts and percentages. Normality was assessed visually using histograms and Q–Q plots.

Between-group comparisons were performed using Student’s *t*-test or one-way analysis of variance for normally distributed continuous variables, and the Mann–Whitney *U* test or Kruskal–Wallis test for non-normally distributed variables. Categorical variables were compared using the χ^2^ test or Fisher’s exact test as appropriate. Correlations between continuous variables were assessed using Spearman’s rank correlation coefficient. Baseline associations between clinical, imaging, and myocardial PET uptake variables were explored using univariable logistic regression. Multivariable logistic regression models were constructed to identify independent predictors of myocardial [^18^F]GP1 uptake, incorporating clinically relevant covariates and imaging measures while avoiding collinearity. Odds ratios are reported with 95% confidence intervals.

Associations between imaging metrics and left ventricular structure and function (ejection fraction and end-diastolic volume) were assessed using linear regression models. Imaging variables were scaled to clinically interpretable units (per 5% increase in late gadolinium enhancement or microvascular obstruction, and per 10% increase in myocardial PET uptake). Multivariable models included infarct burden and microvascular injury parameters as primary mechanistic covariates. Model assumptions were evaluated using residual diagnostics, and collinearity was assessed using variance inflation factors.

Longitudinal analyses evaluated changes in ejection fraction and end-diastolic volume between baseline and follow-up, expressed as absolute change. Associations between baseline imaging metrics and changes in ventricular parameters were assessed using linear regression. Persistence of myocardial PET signal was examined by linear regression of baseline vs. follow-up myocardial PET uptake in patients with paired imaging.

For segmental analyses, myocardial PET uptake and cardiac MR parameters were summarized by myocardial segment and visualized using polar maps. Where applicable, within-patient comparisons and mixed-effects models with random intercepts for patient were used to account for clustering of segments within individuals. All statistical tests were two-sided, and a *P*-value <0.05 was considered statistically significant.

## Results

### Histological correlation of [^18^F]GP1 uptake in human myocardium

*Ex vivo* autoradiography demonstrated focal [^18^F]GP1 binding within infarcted myocardial tissue, corresponding to regions of structural injury and microvascular obstruction. In contrast, remote non-infarcted myocardium showed minimal tracer binding (*Figure 1*). Infarcted regions demonstrated microvascular thrombus formation with fibrin deposition on Martius Scarlet Blue staining together with CD41-positive platelet accumulation. The remote myocardium exhibited preserved myocardial architecture, and absence of microthrombi or CD41 staining (*Figure 1*).

**Figure 1:**
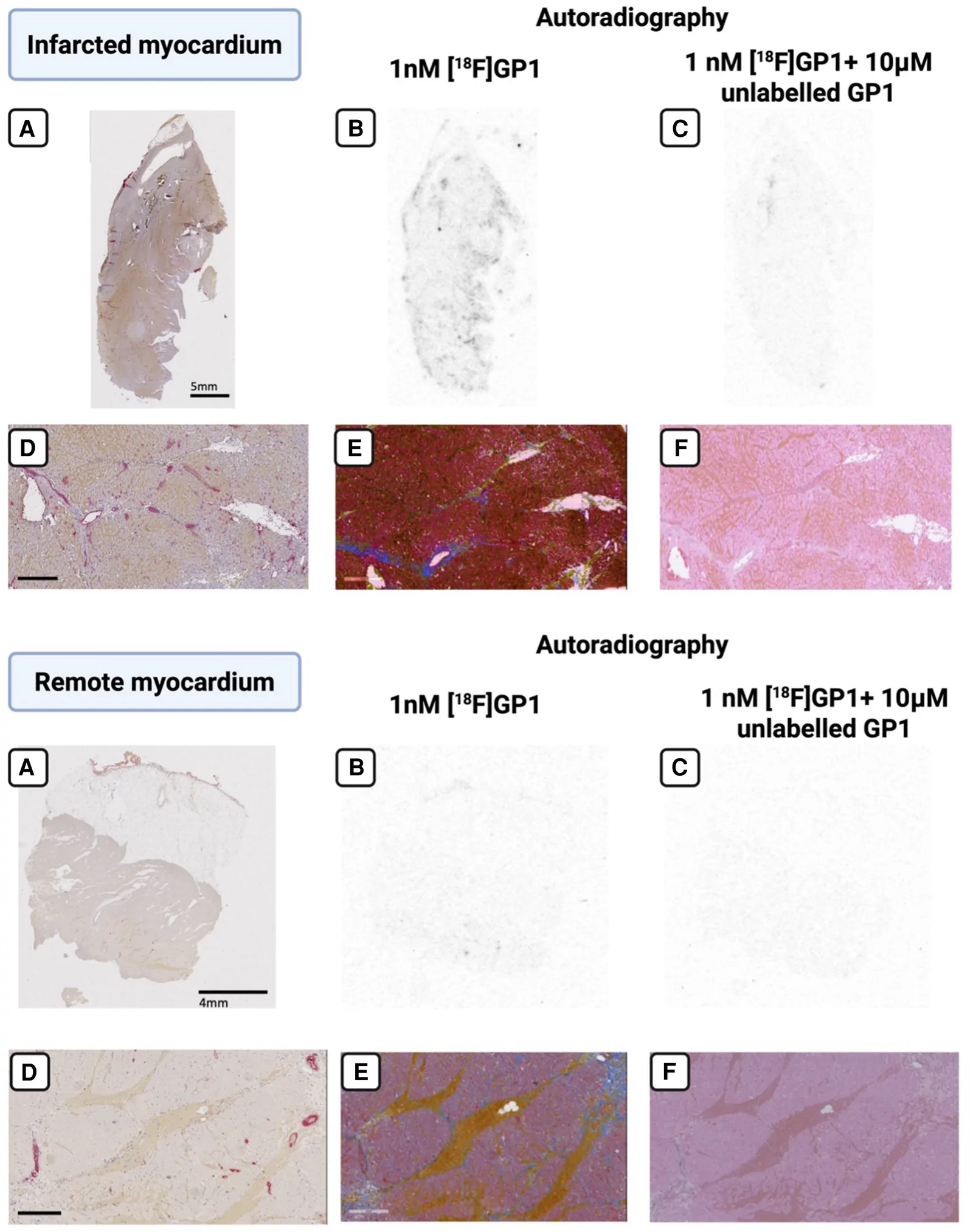
Ex vivo [^18^F]GP1 binding in infarcted vs. remote myocardium. Representative ex vivo myocardial tissue sections demonstrating differential [^18^F]GP1 binding and corresponding histopathological findings in infarcted and remote myocardium. **Top panels** (infarcted myocardium): Panel A, gross myocardial section (scale bar 5 mm); Panel B, autoradiography demonstrating focal [^18^F]GP1 uptake within infarcted regions; Panel C, autoradiographic signal is markedly reduced following competitive inhibition with unlabelled GP1, confirming specific binding to activated glycoprotein IIb/IIIa receptors. Histological analysis demonstrates Panel D, CD41 immunohistochemistry showing platelet accumulation within the microvasculature (brown staining); Panel E, Martius Scarlet Blue staining identifying fibrin deposition (red) and microvascular thrombosis; and Panel F, haematoxylin and eosin staining demonstrating structural disruption consistent with infarcted myocardium. **Bottom panels** (remote myocardium): Panel A, gross myocardial section (scale bar 4 mm); Panel B, autoradiography showing absent [^18^F]GP1 uptake; Panel C, autoradiographic signal following competitive inhibition with unlabelled GP1. Histological analysis demonstrates Panel D, CD41 immunohistochemistry; Panel E, Martius Scarlet Blue staining; and Panel F, haematoxylin and eosin staining, collectively showing no significant platelet accumulation, fibrin deposition, or structural disruption in remote myocardium.

### Study population

One hundred patients (mean age 60 ± 10 years, 91% male) with recent anterior ST-segment elevation myocardial infarction were recruited and underwent [^18^F]GP1 PET/MR imaging at a median time of 13 [9–18] days following index presentation. In all patients, the culprit vessel was the left anterior descending coronary artery which underwent revascularization with primary percutaneous intervention. Normal epicardial coronary flow (TIMI 3 flow) was restored in most patients although reduced flow (TIMI 2 flow) and no-reflow phenomenon (TIMI 0/1 flow) occurred in 19% and 10%, respectively. The rate of peri-procedural intravenous glycoprotein IIb/IIIa receptor antagonist use was 27% (*Table 1*).

**Table 1:** Baseline characteristics of study population and those with and without evidence of myocardial [^18^F]GP1 uptake on positron emission tomography

	*n* = 100	No myocardial [^18^F]GP1 uptake *n* = 56^a^	Myocardial [^18^F]GP1 uptake *n* = 44^a^	*P* value^b^
**Age**	60 ± 10)	62 ± 10)	58 ± 10	0.084
**Sex**				0.727
Male	91 (91)	50 (89)	41 (93)	
Female	9 (9)	6 (11)	3 (7)	
**Body mass index** (kg/m^2^)	25 ± 7	26 ± 9	25 ± 4	0.692
**Time to revascularization** (hours)	14 ± 28	7 ± 9	21 ± 40	**0.006**
**Time to scan** (days)	15 ± 10	17 ± 11	13 ± 8	**0.033**
**Smoking status**				0.831
Current	27 (27)	15 (27)	12 (27)	
Ex-smoker	18 (18)	9 (16)	9 (20)	
Non-smoker	55 (55)	32 (57)	23 (52)	
**Medical history**				
Coronary artery disease	11 (11)	5 (8.9)	6 (14)	0.529
Previous myocardial infarction	9 (9)	5 (8.9)	4 (9.1)	>0.999
Angina	7 (7)	4 (7.1)	3 (6.8)	>0.999
Hypertension	19 (19)	12 (21)	7 (16)	0.485
Hypercholesterolaemia	42 (42)	23 (41)	19 (43)	0.832
Diabetes mellitus	17 (17)	8 (14)	9 (20)	0.415
Peripheral vascular disease	1 (1)	1 (1.8)	0 (0)	>0.999
**Prior procedures**				
Percutaneous coronary intervention	7 (7)	3 (5.4)	4 (9.1)	0.696
Coronary artery bypass grafting	0 (0)			
**Medications**				
Aspirin	99 (99)	56 (100)	43 (98)	0.440
P2Y12 antagonist	100 (100)	56 (100)	44 (100)	
Statin	98 (98)	54 (96)	44 (100)	0.502
Beta blocker	92 (92)	50 (89)	42 (95)	0.460
Angiotensin-converting enzyme inhibitor or angiotensin receptor antagonist	97 (97)	54 (96)	43 (97)	>0.999
Mineralocorticoid receptor antagonist	26 (26)	9 (16)	17 (38)	**0.011**
Insulin	3 (3)	0 (0)	3 (6.8)	0.082
Oral diabetic medications	16 (16)	9 (16)	7 (16)	0982
Tirofiban	27 (27)	15 (27)	12 (27)	0.957
Anticoagulation	17 (17)	2 (4)	15 (34)	**<0.001**
**Persistent ST-segment elevation on electrocardiogram**	41 (41)	15 (27)	26 (59)	**0.001**
**Left ventricular ejection fraction on echocardiography** (%)	44 ± 9	47 ± 8	41 ± 8	**0.002**
**Left ventricular ejection fraction on cardiac MR (%)**	47 ± 11	52 ± 10	40 ± 8	**<0.001**
**Left ventricular end diastolic volume** (**mL)**	88 ± 18	82 ± 19	95 ± 15	**<0.001**
**Presence of microvascular obstruction on cardiac MR**	40 (40)	0 (0)	40 (91)	**<0.001**
**Infarct burden (% late gadolinium enhancement)**	20 ± 14	12 ± 10	29 ± 11	**<0.001**
**Burden of microvascular obstruction on cardiac MR (%)**	2.9 ± 4.3	0 ± 0	6.5 ± 4.3	**<0.001**
**Burden of myocardial platelet activation (%)**	11 ± 15	0 ± 0	23 ± 14	**<0.001**
**TBRmax**	1.27 ± 0.68	0.75 ± 0.24	1.89 ± 0.5	**<0.001**
**SUVmax**	2.18 ± 1.13	1.43 ± 0.52	3.08 ± 0.99	**<0.001**
**No-reflow phenomenon**	40 (40)	5 (9)	35 (80)	**<0.001**
**TIMI flow following revascularization**				0.072
0	2 (2)	1 (2)	1 (2)	
1	8 (8)	2 (4)	6 (14)	
2	19 (19)	8 (14)	11 (25)	
3	71 (71)	45 (80)	26 (59)	
**Cerebral infarction on brain MR**	15 (15)	5 (9)	10 (23)	0.055
**Left ventricular thrombus**	30 (30)	4 (7)	26 (59)	**<0.001**

^a^Data are presented as *n* (%) or mean ± standard deviation.

^b^P values derived using Wilcoxon rank-sum test for continuous variables, Pearson's chi-squared test for categorical variables, and Fisher's exact test where expected cell counts were less than five.

BMI, body-mass index; CABG, coronary artery bypass grafting; CI, cerebral infarction; ECG, electrocardiogram; LV, left ventricle; LVEDD, left ventricular end-diastolic diameter; LVEF, left ventricular ejection fraction; MR, magnetic resonance; MVO, microvascular obstruction; PCI, percutaneous coronary intervention; ST, ST-segment; SUVmax, maximum standardized uptake value; TBRmax, maximum target-to-background ratio; TIMI, thrombolysis in myocardial infarction.

### Myocardial [^18^F]GP1 uptake

In total, 132 hybrid [^18^F]GP1 PET/MRI scans were performed in 100 patients: 100 scans at baseline and 32 repeat scans at 3–6 months. Administration of [^18^F]GP1 was well tolerated with no participants reporting any immediate or short-term adverse reactions. Mean radiation exposure was 5.3 ± 0.2 mSv. The mean left ventricular ejection fraction was 47 ± 11% and cardiac MR-defined microvascular obstruction was present in 40% of patients.

Myocardial [^18^F]GP1 uptake was observed in 44% of patients (TBR_max_ 1.89 ± 0.50), while 56% demonstrated no myocardial [^18^F]GP1 uptake (TBR_max_ 0.75 ± 0.24; *P* < 0.001). Full reproducibility statistics and Bland–Altman plots are presented in Supplementary data online, *Tables S1*  to  *S4*. All participants with cardiac MR-defined microvascular obstruction also demonstrated increased myocardial [^18^F]GP1 uptake. Four patients demonstrated increased myocardial [^18^F]GP1 uptake, with no underlying evidence of microvascular obstruction on MR. Diagnostic performance analysis demonstrated 100% sensitivity and 93% specificity, corresponding to an overall accuracy of 96% for myocardial [^18^F]GP1 uptake in detecting cardiac MR-defined microvascular obstruction *(Figure 2)*.

**Figure 2:**
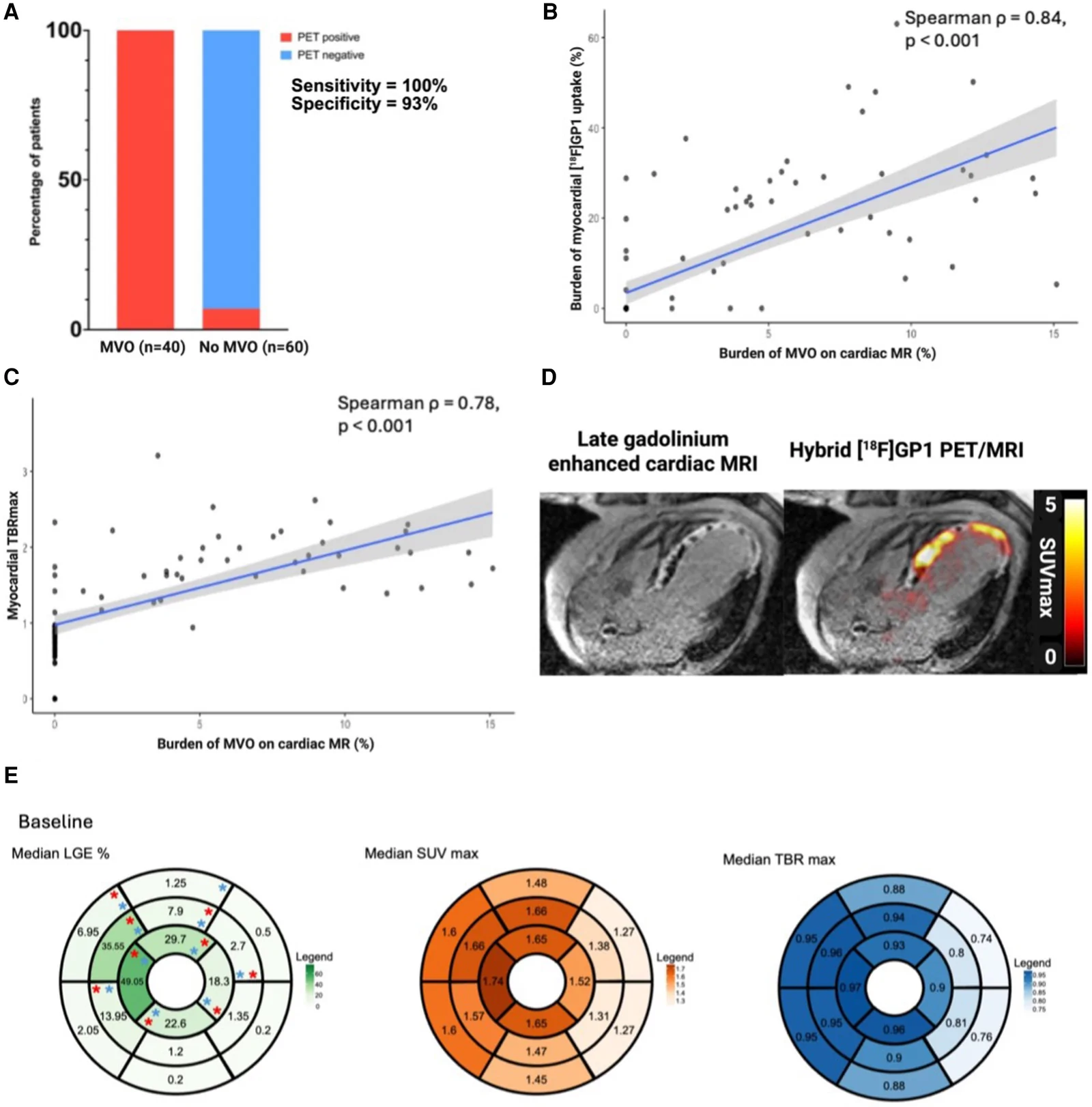
Association between myocardial [^18^F]GP1 uptake and microvascular obstruction (MVO). (*A*) Diagnostic performance of myocardial [^18^F]GP1 positron emission tomography for detection of cardiac magnetic resonance (MR) defined microvascular obstruction. All patients with MVO demonstrated myocardial [^18^F]GP1 uptake, resulting in 100% sensitivity and 93% specificity for detecting MVO. **(***B***)** Relationship between burden of myocardial [^18^F]GP1 uptake and burden of MVO on cardiac MR imaging. **(***C***)** Association between myocardial [^18^F]GP1 uptake intensity, quantified as TBRmax, and MVO burden. **(***D***)** Representative hybrid [^18^F]GP1 PET/MRI images demonstrating focal myocardial tracer uptake co-localizing with infarcted myocardium identified on late gadolinium enhancement and extending beyond regions of cardiac MR defined areas of microvascular obstruction. **(***E***)** Polar maps based on the American Heart Association 17-segment left ventricular model showing median regional late gadolinium enhancement (LGE), myocardial maximum standardized uptake value (SUVmax), and target-to-background ratio (TBRmax) at baseline. Segments are displayed according to standard left ventricular anatomy, with anterior and anteroseptal territories corresponding to the left anterior descending coronary artery distribution. At baseline, myocardial [^18^F]GP1 uptake co-localizes with infarct territories but demonstrates a broader spatial distribution than LGE and regions of microvascular obstruction, indicating platelet activity extending beyond structurally defined cardiac MR injury.

Compared to those without uptake, patients with myocardial [^18^F]GP1 uptake at baseline exhibited more severe myocardial injury, including lower baseline left ventricular ejection fraction, larger end-diastolic volumes, greater infarct size, and higher prevalence of no-reflow (all *P* < 0.001). Time from symptom onset to revascularization (7 ± 9 vs. 21 ± 40 h, respectively, *P* = 0.006) and time from symptom onset to imaging (17 ± 11 vs. 13 ± 8 days, respectively, *P* = 0.033) differed between these two groups.

The regional distribution of myocardial [^18^F]GP1 uptake was consistent with the territory supplied by the left anterior descending coronary artery. Segmental analysis demonstrated close spatial concordance between myocardial [^18^F]GP1 uptake and regions of infarction by late gadolinium enhancement (*Figure 2*). Notably, myocardial [^18^F]GP1 uptake was not observed outside of areas of myocardial infarction. Whilst the distribution of myocardial platelet activation mirrored the pattern of microvascular obstruction on cardiac MR, the burden exceeded cardiac MR-defined burden of microvascular obstruction by a median difference of 18 [8 to 22] %. Furthermore, segments with greater late gadolinium enhancement burden exhibited correspondingly higher myocardial SUV_max_ and TBR_max_ values.

### Determinants of myocardial [^18^F]GP1 uptake

Microvascular obstruction burden on cardiac MR correlated strongly with myocardial [^18^F]GP1 uptake intensity (Spearman ρ = 0.78; *P* < 0.001) and burden (Spearman ρ = 0.84; *P* < 0.001) independent of infarct size (percentage late gadolinium enhancement burden) (*Figure 2*). In univariable linear regression, each 1% increase in microvascular obstruction on cardiac MR was associated with a 0.10 increase in myocardial TBR_max_ (95% CI 0.07–0.12; *P* < 0.001). After adjustment for infarct size, microvascular obstruction on cardiac MR remained independently associated with myocardial [^18^F]GP1 TBR_max_ (β = 0.048 per 1% microvascular obstruction, 95% CI 0.016–0.080; *P* = 0.003).

On univariable logistic regression, myocardial [^18^F]GP1 uptake was associated with lower left ventricular ejection fraction, greater end-diastolic volume, greater infarct burden, and angiographic no-reflow (all *P* < 0.001; *Table 2*). In multivariable models incorporating measures of infarct size and microvascular injury, angiographic no-reflow demonstrated the strongest association (adjusted odds ratio 16.15, 95% confidence interval 3.61 to 72.22, *P* < 0.001) with myocardial [^18^F]GP1 uptake. In the fully adjusted model, only angiographic no-reflow remained independently associated with myocardial [^18^F]GP1 uptake (*Table 2*). Full multivariable model results including sensitivity analyses and variance inflation factors are presented in Supplementary data online, *Tables S5*  to  *S11*.

**Table 2:** Associations with myocardial [^18^F]GP1 uptake

Variable	Univariable OR (95% CI)	*P* value	Model 1^a^ OR (95% CI)	*P* value	Model 2^b^ OR (95% CI)	*P* value
**Cardiac structure and function**						
Ejection fraction on cardiac MR (%)	0.87 (0.83 to 0.93)	**<0.001**	1.03 (0.93 to 1.14)	0.622	0.99 (0.91 to 1.09)	0.986
End-diastolic volume on cardiac MR (mL)	1.05 (1.02 to 1.08)	**<0.001**	1.05 (1.00 to 1.11)	0.068		
Degree of transmural late gadolinium enhancement (No LGE, 1% to 25%, 26% to 50%, 51 to 75% and 76 to100%)	8.93 (3.78 to 21.11)	**<0.001**	4.99 (1.89 to 13.21)	**0.001**	2.75 (0.95 to 7.99)	0.063
Infarct burden (% late gadolinium enhancement - per %)	1.16 (1.10 to 1.23)	**<0.001**			1.08 (0.99 to 1.18)	0.084
**Microvascular obstruction**						
Persistent ST-segment elevation	3.95 (1.70 to 9.17)	**0.001**	1.62 (0.35 to 7.56)	0.537		
No-reflow on coronary angiography	39.67 (12.25 to 128.42)	**<0.001**	23.15 (4.46 to 120.11)	**<0.001**	16.15 (3.61 to 72.22)	**<0.001**
**Clinical covariates**						
Age	0.96 (0.93 to 1.01)	0.087				
Diabetes mellitus	1.54 (0.54 to 4.39)	0.417				
Smoking	1.17 (0.70 to 1.95)	0.551				
Hypercholesterolaemia	1.09 (0.49 to 2.43)	0.832				
Hypertension	0.69 (0.25 to 1.94)	0.486				
Time to revascularization (per hour)	1.03 (1.00 to 1.07)	0.058				
Prior myocardial infarction	1.02 (0.26 to 4.05)	0.978				
Prior percutaneous coronary intervention	1.77 (0.37 to 8.34)	0.472				
Tirofiban use	1.03 (0.42 to 2.49)	0.957				

^a^Model 1 (Core imaging model): Adjusted for ejection fraction, end-diastolic volume, degree of late gadolinium enhancement, persistent ST-segment elevation, and angiographic no-reflow.

^b^Model 2 (Fully adjusted model): Adjusted for degree of late gadolinium enhancement, angiographic no-reflow, and key clinical covariates retained based on biological plausibility and model stability.

Odds ratios are per unit increase unless otherwise stated. Microvascular obstruction was perfectly concordant with myocardial [^18^F]GP1 uptake and was therefore not included in logistic regression models.

CI, confidence interval; LGE, late gadolinium enhancement; MR, magnetic resonance; MVO, microvascular obstruction; OR, odds ratio; PCI, percutaneous coronary intervention; ST, ST-segment; TIMI, Thrombolysis in Myocardial Infarction.

At baseline, greater burden of myocardial platelet activation was associated with lower left ventricular ejection fraction and larger end-diastolic volume on univariable linear regression. However, after adjustment for infarct burden (late gadolinium enhancement) and microvascular obstruction, myocardial PET measures were no longer independently associated with ventricular function. In contrast, infarct size remained a strong independent determinant of both ejection fraction and end-diastolic volume (*Table 3*).

**Table 3:** Imaging predictors of left ventricular function

Left ventricular ejection fraction (%)					
	Unit	Univariable β (95% CI)	*P* value	Multivariable^a^ β (95% CI)	*P* value
**Myocardial PET uptake**					
Burden of myocardial platelet activation (%)	Per 10%	−3.88 (−5.13 to −2.64)	<0.001	−0.69 (−2.29 to 0.91)	0.394
TBRmax	Per unit	−8.74 (−11.34 to −6.14)	<0.001	−1.60 (−5.06 to 1.86)	0.360
SUVmax	Per unit	−4.24 (−5.93 to −2.55)	<0.001	−1.02 (−2.90 to 0.86)	0.284
**Infarct burden**					
Infarct burden (% Late gadolinium enhancement)	Per 5%	−2.78 (−3.35 to −2.20)	<0.001	−2.16 (−3.04 to −1.28)	**<0**.**001**
**Microvascular injury**					
Burden of microvascular obstruction on cardiac MR (%)	Per 5%	−7.17 (−9.26 to −5.08)	<0.001	−1.15 (−4.19 to 1.89)	0.454
Angiographic no-reflow	Present vs. absent	−10.18 (−14.14 to −6.21)	<0.001	−1.08 (−6.14 to 3.98)	0.673

Left ventricular end-diastolic volume (mL)					
Parameter	Unit	Univariable β (95% CI)	*P* value	Multivariable^a^ β (95% CI)	*P* value
**Myocardial PET uptake**					
Burden of myocardial platelet activation (%)	Per 10%	3.84 (1.48 to 6.20)	0.002	0.46 (−2.90 to 3.83)	0.785
TBRmax	Per unit	10.42 (5.47 to 15.37)	<0.001	6.17 (−1.32 to 13.66)	0.105
SUVmax	Per unit	4.23 (0.99 to 7.48)	0.012	0.97 (−3.06 to 4.99)	0.634
**Infarct burden**					
Infarct burden (% late gadolinium enhancement)	Per 5%	3.21 (2.01 to 4.40)	<0.001	2.62 (0.70 to 4.53)	**0**.**008**
**Microvascular injury**					
Burden of microvascular obstruction on cardiac MR (%)	Per 5%	6.84 (2.78 to 10.91)	0.001	0.38 (−6.20 to 6.95)	0.910
Angiographic no-reflow	Present vs. absent	9.11 (1.80 to 16.41)	0.015	−6.03 (−16.98 to 4.92)	0.277

Linear regression analyses of myocardial [^18^F]GP1 metrics and imaging correlates with left ventricular ejection fraction and end-diastolic volume.

^a^Multivariable models adjusted for late gadolinium enhancement, microvascular obstruction, angiographic no-reflow, and the specified myocardial [^18^F]GP1 metric.

Regression coefficients represent absolute change in left ventricular ejection fraction (%) or end-diastolic volume (mL) per unit increase in the predictor.

β, unstandardized regression coefficient; CI, confidence interval; EDV, end-diastolic volume; EF, ejection fraction; LGE, late gadolinium enhancement; MR, magnetic resonance; MVO, microvascular obstruction; PET, positron emission tomography; SUVmax, maximum standardized uptake value; TBRmax, maximum target-to-background ratio.

### Persistence of myocardial [^18^F]GP1 uptake

Amongst the 32 patients with follow-up myocardial [^18^F]GP1 imaging (median time interval 125 [100–172] days), 7 patients (22%) demonstrated persistent myocardial [^18^F]GP1 uptake and cardiac MR-defined microvascular obstruction at follow up. Resolution of cardiac MR-defined microvascular obstruction was observed in 25 patients (78%), which correlated with absence of myocardial [^18^F]GP1 uptake. The spatial distribution of myocardial [^18^F]GP1 uptake remained concordant with baseline infarct territories, with persistent regional myocardial [^18^F]GP1 uptake observed despite relative stability of late gadolinium enhancement distribution.

Baseline myocardial [^18^F]GP1 uptake was associated with persistence of myocardial [^18^F]GP1 uptake at follow-up, such that each 10% increase in baseline [^18^F]GP1 burden was associated with a 4% greater burden at follow-up (β = 3.63, *P* = 0.007), with no other baseline imaging or clinical variables demonstrating independent associations. In contrast, baseline burden of myocardial [^18^F]GP1 uptake demonstrated no association with follow-up infarct burden (β = + 2.2% per 10% PET, *P* = 0.15), whilst baseline late gadolinium enhancement remained the dominant determinant of follow-up infarct burden (adjusted R^2^ = 0.75; *P* < 0.0001).

Baseline myocardial [^18^F]GP1 uptake was not associated with change in ejection fraction or change in end-diastolic volume on univariable analysis (*R* = 0.17, *P* = 0.37 and *R* = 0.05, *P* = 0.79, respectively) irrespective of whether uptake was quantified as burden of myocardial platelet activation, SUV_max_ or TBR_max_. These findings remained unchanged after adjustment for baseline ventricular measures, infarct burden, and microvascular obstruction. At follow-up, left ventricular ejection fraction increased by a median of approximately 4%, while end-diastolic volume demonstrated modest variability across patients. Patients with persistent [^18^F]GP1 uptake had lower left ventricular ejection fraction at follow-up compared with those in whom [^18^F]GP1 uptake had resolved (39% vs. 46%, *P* = 0.02) (*Figure 3*). End-diastolic volume at follow-up did not differ between the groups (103 vs. 94 mL, *P* = 0.29).

**Figure 3:**
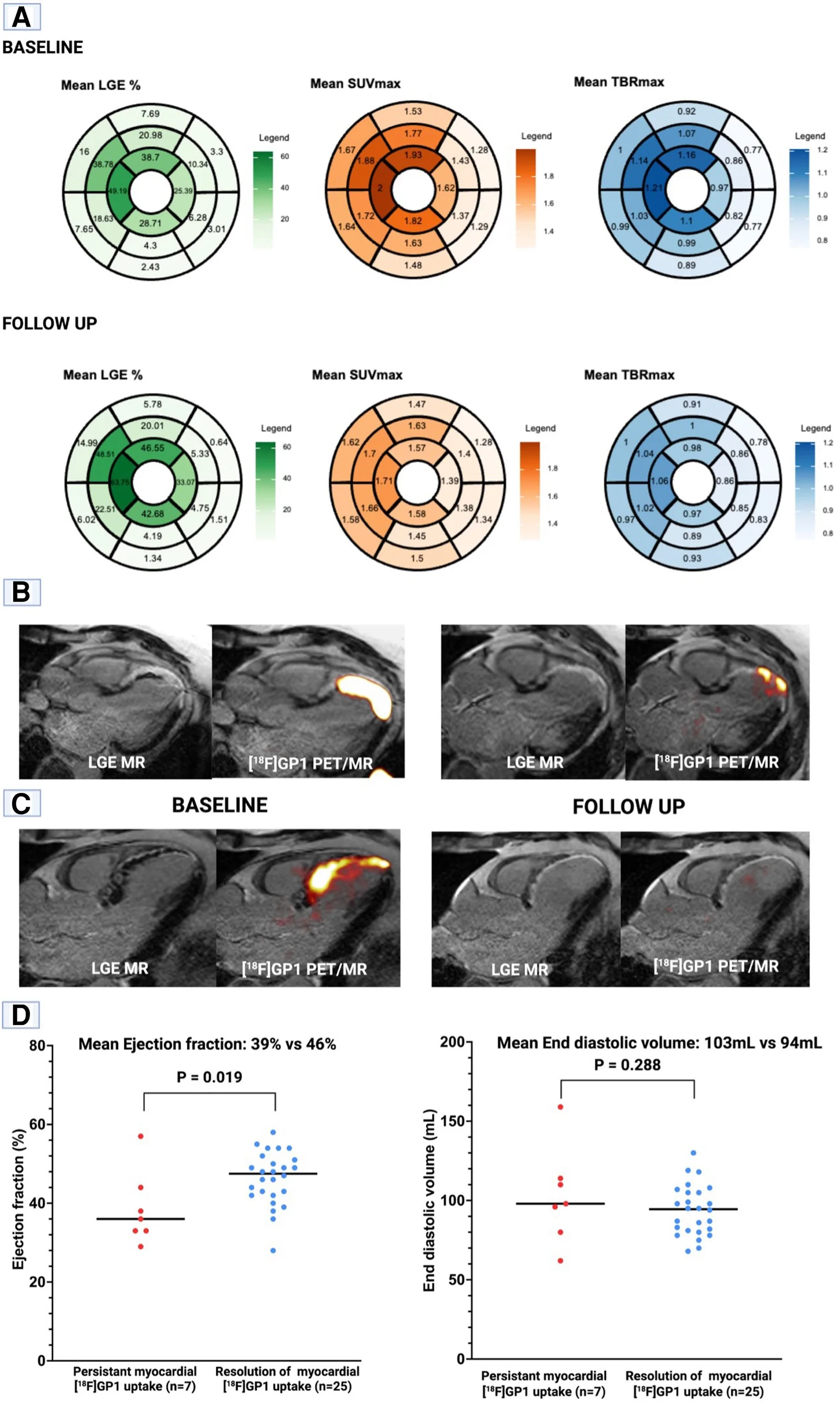
Segmental distribution of myocardial [^18^F]GP1 uptake and infarct burden at baseline and follow-up. (*A*) Polar maps based on the American Heart Association 17-segment left ventricular model showing mean regional late gadolinium enhancement (LGE), myocardial maximum standardized uptake value (SUVmax), and target-to-background ratio (TBRmax) at baseline (top row) and follow-up (bottom row) in patients who underwent repeat imaging (*n* = 32). Segments are displayed according to standard left ventricular anatomy. At baseline, myocardial [^18^F]GP1 uptake co-localizes with infarct territories and increases with infarct burden. At follow-up, regional LGE remains largely unchanged, while persistent focal [^18^F]GP1 signal is observed within infarcted myocardium, indicating ongoing platelet-related biological activity. (*B*) Representative hybrid [^18^F]GP1 PET/MR images demonstrating myocardial tracer uptake at baseline with persistent focal uptake at follow-up, consistent with ongoing platelet activation within infarcted myocardium. (*C*) Representative hybrid [^18^F]GP1 PET/MR images demonstrating myocardial tracer uptake at baseline with resolution of uptake at follow-up, illustrating variability in the temporal evolution of platelet-related signal. (*D*) Left ventricular ejection fraction at follow-up according to persistence of myocardial [^18^F]GP1 uptake. Patients with persistent uptake demonstrated lower ejection fraction compared with those in whom uptake had resolved (mean 39% vs. 46%, *P* = 0.02), with no significant difference in end-diastolic volume. Each point represents an individual patient, with horizontal bars indicating group means. Colour scales represent mean values for each parameter within each segment.

## Discussion

In this prospective hybrid PET/MR study of patients with recent anterior ST-segment elevation myocardial infarction, we provide the first direct *in vivo* evidence that microcirculatory platelet activation and thrombosis are universally associated with microvascular obstruction. Myocardial [^18^F]GP1 uptake extended beyond regions of cardiac MR-defined microvascular obstruction and was identified in a small subset of patients without detectable microvascular obstruction. The magnitude of microcirculatory platelet activation and thrombosis was proportional to infarct severity and was higher in patients with no-reflow or more delayed reperfusion. In a subset of patients, myocardial [^18^F]GP1 uptake persisted out to 3–6 months, suggesting ongoing microvascular thrombosis beyond the acute phase. Resolution of cardiac MR-defined microvascular obstruction at follow-up was universally concordant with resolution of myocardial [^18^F]GP1 uptake, providing prospective longitudinal evidence that platelet activation tracks structural microvascular recovery. Together, these findings demonstrate that myocardial platelet activation is universally and quantitatively associated with microvascular obstruction, consistent with an important mechanistic contribution of platelet-mediated thrombosis to no-reflow and microvascular injury and highlight a potentially targetable biological pathway in a condition for which effective therapies remain lacking.

Microvascular obstruction remains a major determinant of infarct size, adverse ventricular remodelling and clinical outcomes following ST-segment elevation myocardial infarction, yet its underlying mechanisms remain incompletely understood.^1,4–6^ Our findings provide the first direct *in vivo* human evidence characterizing platelet activation and platelet-mediated thrombosis as an important mechanistic contributor to the pathogenesis of microvascular obstruction. Importantly, complementary illustrative evidence from *ex vivo* histological analyses demonstrated both platelets and fibrin within microthrombi of the infarcted myocardium. However, our studies cannot rule out direct platelet activation within the microcirculation due to endothelial disruption, exposure of the subendothelial matrix and local thrombo-inflammatory signalling. This could lead to *in situ* platelet adhesion, aggregation and interaction with leukocytes within the microcirculation itself. These processes may result in microvascular plugging and amplification of microvascular injury independent of, or in addition to, distal embolization. We acknowledge that the alternative interpretation that GPIIb/IIIa-mediated platelet activation is reactive and secondary to downstream ischaemic tissue injury and this cannot be excluded from our observational dataset.

We observed a strong relationship between time to reperfusion with patients demonstrating both [^18^F]GP1 uptake and microvascular obstruction having substantially longer ischaemic times. Following initial formation, thrombus undergoes progressive structural stabilization and organization with cross-linking of fibrin which ensures it becomes increasingly resistant to thrombus dissolution and degradation. Taken together with our histological findings, these observations support a mechanistic model in which proximal thrombus resistant to dissolution embolizes and occludes the myocardial microcirculation to cause microvascular obstruction. This is consistent with the strong correlation with no-reflow phenomenon during primary percutaneous coronary intervention. This mechanistic framework is directly supported by the T-TIME ischaemic time subgroup analysis, which demonstrated that adjunctive thrombus-targeting therapy increased microvascular obstruction in patients presenting at ≥4–6 h, attributed to progressive fibrin cross-linking that renders established thrombus fragmentation resistant to dissolution.^25^ A recent comprehensive narrative review identifies a therapeutic window of 60–180 min from symptom onset, noting that beyond 4–6 h the myocardium within the area at risk has typically undergone irreversible necrosis, limiting the efficacy of any intervention.^7^ Patients in our cohort who had myocardial [^18^F]GP1 uptake had markedly longer ischaemic times than those without uptake (21 ± 40 vs. 7 ± 9 h), consistent with greater thrombus organization and stabilization in those with established microvascular obstruction.

The clinical relevance of GPIIb/IIIa-mediated platelet activation in microvascular obstruction is directly supported by randomized trial evidence. The REVERSE-FLOW trial evaluated guideline-recommended bailout GPIIb/IIIa receptor antagonism in 120 patients with angiographic microvascular obstruction and no-reflow (TIMI flow ≤2 after primary percutaneous coronary intervention).^26^ GPIIb/IIIa receptor antagonism reduced both the prevalence and extent of cardiac MR-defined microvascular obstruction, without reducing the primary endpoint of infarct size and at the cost of increased non-life-threatening bleeding. In an exploratory post-hoc analysis restricted to patients with coronary occlusion (TIMI 0/1 flow after PCI), GPIIb/IIIa receptor antagonism did demonstrate a reduction in infarct size (17.0% vs. 33.7%, *P* = 0.034). This group of patients are consistent with the clinical phenotype most strongly associated with myocardial [^18^F]GP1 uptake in our cohort. In contrast, in the PITRI trial, intensification of P2Y12 inhibition with intravenous cangrelor added to oral ticagrelor at the time of reperfusion in 210 patients with ST-segment elevation myocardial infarction produced no reduction in infarct size, no reduction in microvascular obstruction prevalence or extent, and no increase in bleeding, despite achieving approximately 2-fold faster platelet inhibition during percutaneous coronary intervention.^27^

The mechanistic contrast and difference in outcomes between REVERSE-FLOW and PITRI is directly informative. The radiotracer [^18^F]GP1 binds with high affinity and specificity to the activated GPIIb/IIIa receptor, which is the final common pathway for platelet aggregation, activated by thrombin, collagen, and von Willebrand factor under shear stress and is independent of P2Y12-mediated ADP signalling. Importantly, whilst GPIIb/IIIa receptor antagonism reduced microvascular obstruction burden in REVERSE-FLOW, additional P2Y12 blockade demonstrated no benefit in PITRI, which is mechanistically consistent with residual platelet activation in established microvascular obstruction being driven through P2Y12-independent pathways, most plausibly through thrombin and collagen-mediated activation from acutely disrupted endothelium and distal atherothrombotic embolization. This aligns with an acute atherothrombotic model of microvascular obstruction and supports the GPIIb/IIIa receptor itself as the mechanistically relevant therapeutic target and remains the biology interrogated by [^18^F]GP1 PET imaging. Furthermore, the fact that REVERSE-FLOW reduced microvascular obstruction burden is consistent with platelet-mediated biology remaining active and partially targetable in the subacute phase, even when irreversible microvascular injury has already occurred. The ability of [^18^F]GP1 PET to quantify active GPIIb/IIIa-engaged thrombosis *in vivo*, rather than inferring it from structural imaging or angiographic appearance, provides a rationale for using molecular imaging to select patients with early active thrombus most likely to benefit from targeted GPIIb/IIIa receptor antagonist therapy, distinguishing them from those with predominantly organized fibrin-dominant thrombus where such strategies may be ineffective or harmful. This represents a specific and testable translational hypothesis warranting prospective evaluation, rather than a current treatment recommendation.

Myocardial [^18^F]GP1 uptake extended beyond regions of microvascular obstruction identified on cardiac MR imaging and was present in a subset of patients without detectable microvascular obstruction. Cardiac MR-defined microvascular obstruction is anatomically defined as it identifies myocardium where capillary obstruction or destruction is severe enough to prevent contrast entry, and by definition captures only the most advanced stage of microvascular injury. In contrast, the radiotracer [^18^F]GP1 images the active biological process of platelet activation and adhesion via the activated GPIIb/IIIa receptor and therefore may be sensitive to microvascular pathology that precedes, or exists independently of, the structural threshold required for microvascular obstruction to become visible on cardiac MR. Notably, patients with myocardial [^18^F]GP1 uptake in the absence of microvascular obstruction demonstrated shorter time to reperfusion but still exhibited impaired left ventricular function and persistent ST-segment elevation, consistent with functionally significant microvascular injury below the structural threshold detectable by gadolinium-based imaging. These observations suggest that platelet-mediated microvascular injury may be more widespread than is apparent using conventional imaging techniques, and that molecular imaging of platelet activation provides a more sensitive assessment of the underlying biological processes underpinning microvascular obstruction. This interpretation is consistent with prior invasive physiology data demonstrating that anatomical and functional measures of microvascular injury are frequently discordant. De Maria *et al.*^28^ have previously reported discordance between the Index of Microcirculatory Resistance and cardiac MR-defined microvascular obstruction in a third of patients with ST-segment elevation myocardial infarction, with only a modest correlation between Index of Microcirculatory Resistance and microvascular obstruction extent. Notably, a small subgroup had abnormal Index of Microcirculatory Resistance despite the complete absence of cardiac MR-defined microvascular obstruction and showed no infarct-size regression over six months, in contrast to patients with normal Index of Microcirculatory Resistance and no microvascular obstruction, who showed regression over the same interval. The authors attributed this to extensive microvascular stunning not captured by cardiac MR, concluding that absence of anatomical microvascular obstruction did not equate to microvascular integrity. Our [^18^F]GP1 findings may extend this principle into the molecular domain. Regions of platelet activation outside the cardiac MR-defined microvascular obstruction core could represent a ‘penumbra’ of microvascular dysfunction, and a territory where myocardial perfusion impairment is sufficient to trigger platelet adhesion and GPIIb/IIIa receptor activation, but has not (or has not yet) progressed to the structural failure required for the detection of microvascular obstruction on cardiac MR. Together, these observations suggest that the burden of microvascular injury may be underestimated using structural imaging approaches alone, and that molecular imaging of platelet activation provides a more sensitive assessment of the underlying biological processes underpinning microvascular obstruction.

The present study includes serial [^18^F]GP1 PET/MR imaging at a median of 125 days in 32 patients, providing prospective longitudinal data directly relevant to this question. Resolution of cardiac MR-defined microvascular obstruction was observed in 25/32 patients (78%), and in every one of these patients, myocardial [^18^F]GP1 uptake also resolved, revealing a complete concordance between structural and molecular recovery. The 7 patients (22%) with persistent cardiac MR-defined microvascular obstruction at follow-up all demonstrated persistent [^18^F]GP1 uptake, with spatial distribution remaining concordant with baseline infarct territories throughout. This bidirectional tracking provides prospective evidence that the additional PET-positive territory observed beyond the cardiac MR-defined microvascular obstruction core at baseline is not an independent or irreversible biological process but rather represents reversible functional microvascular dysfunction that resolves in parallel with structural microvascular injury. These findings support the penumbral interpretation that [^18^F]GP1 PET detected platelet activation extending beyond anatomically defined microvascular obstruction reflects the outer margin of a biologically unified process, rather than a distinct pathological entity. Larger, prospectively powered serial imaging studies are needed to test this hypothesis definitively and to determine whether early quantification of penumbral PET burden predicts the trajectory of structural microvascular obstruction.

Quantitative measures of myocardial [^18^F]GP1 uptake correlated strongly with microvascular obstruction burden and angiographic no-reflow, consistent with a close biological relationship between platelet activation and both structural and functional manifestations of microvascular injury. Importantly, after adjustment for infarct size and microvascular obstruction burden, myocardial [^18^F]GP1 uptake was not an independent determinant of left ventricular ejection fraction or end-diastolic volume, indicating that in this cross-sectional analysis, [^18^F]GP1 PET uptake tracks with infarct severity rather than providing incremental prognostic information beyond established cardiac MR markers. The clinical value of [^18^F]GP1 PET in this context therefore lies in mechanistic characterization and potential therapeutic stratification rather than independent risk prediction. Baseline myocardial [^18^F]GP1 uptake predicted persistence of uptake at follow-up, and patients with persistent uptake had lower left ventricular ejection fraction at follow-up: an exploratory finding that warrants further prospective evaluation in larger studies. Persistent cardiac MR-defined microvascular obstruction, as observed in 22% of patients at follow-up in the present study, has been associated with adverse ventricular remodelling, intramyocardial haemorrhage and worse clinical outcomes.^29^ Microvascular thrombosis may persist beyond the acute reperfusion phase in a small subset of patients, representing a potential contributor to impaired functional recovery, although the mechanisms underlying persistent signal at this timepoint require further dedicated study.

Our *ex vivo* analyses demonstrated spatial concordance between [^18^F]GP1 binding and platelet-rich microvascular thrombosis within infarcted myocardium, with minimal tracer binding and platelet accumulation in remote non-infarcted tissue, providing complementary illustrative support for the biological specificity of [^18^F]GP1 for activated platelet-mediated microvascular thrombosis. Competitive blockade by co-incubation with excess unlabelled GP1 provides mechanistic evidence of specific GPIIb/IIIa binding rather than non-specific tracer retention within infarcted tissue. However, localization of signal specifically to platelet-rich microthrombi within microvascular obstruction territories cannot be confirmed from *ex vivo* tissue sections alone, and non-specific retention, thrombo-inflammatory platelet activation, or endothelial dysfunction may contribute to the *in vivo* signal in ways that cannot be fully excluded from this dataset.

Our findings have important clinical implications. Despite timely reperfusion and contemporary antiplatelet therapy, a substantial proportion of patients develop microvascular obstruction for which no proven effective therapies exist. Previous clinical trials targeting microvascular obstruction have yielded inconsistent or neutral results, likely reflecting the complex and multifactorial nature of the underlying pathophysiology.^7^ The present study provides a mechanistic basis for understanding this therapeutic gap and a rationale for patient stratification in future trials.

This study has several limitations. We acknowledge that [^18^F]GP1 PET requires cyclotron-produced radiopharmaceutical production, hybrid PET/MR hardware, radiation exposure, and substantially greater cost and logistical complexity than cardiac MR alone, and its current role is as a mechanistic research tool rather than a clinical diagnostic tool. It was conducted in a single centre with a modest sample size, and findings require validation in larger multicentre cohorts. The cohort comprised exclusively anterior ST-segment elevation myocardial infarction, and whether these findings generalize to non-anterior infarct territories or to female patients requires dedicated future study. Imaging was performed at a subacute timepoint at a median of 13 days following infarction, reflecting practical constraints of hybrid PET/MR scheduling and radiotracer production, and earlier imaging would capture a biologically distinct and arguably more directly relevant phase of microvascular thrombosis, and represents an important direction for future work. Although [^18^F]GP1 binding is highly specific for activated platelets, the spatial resolution of PET imaging may limit precise localization at the microvascular level, and non-specific retention, inflammatory activity or haemorrhage-associated processes cannot be entirely excluded as contributors to the *in vivo* signal. T2*-weighted imaging for intramyocardial haemorrhage characterization was not performed as part of this pre-specified protocol. The mechanistic relationship between intramyocardial haemorrhage and myocardial [^18^F]GP1 uptake represents an important unresolved question for future dedicated studies. Finally, causality cannot be established from this observational study, and the possibility that myocardial platelet activation represents a downstream marker of ischaemic tissue injury rather than a primary driver of microvascular obstruction cannot be excluded.

In conclusion, myocardial platelet activation is universally associated with microvascular obstruction following ST-segment elevation myocardial infarction, extending beyond structural abnormalities identified by cardiac MR and resolving in complete concordance with structural microvascular recovery. These findings are consistent with an important mechanistic contribution of activated platelet-mediated thrombosis to no-reflow and microvascular injury, arising from both distal embolization of organizing thrombus and *in situ* thrombo-inflammatory processes within the microcirculation. Together, these findings provide a mechanistic basis for understanding the limited success of prior therapeutic strategies and highlight the potential of molecular imaging of platelet activation to identify patients most likely to benefit from targeted GPIIb/IIIa receptor antagonist therapy in a condition for which effective treatments remain elusive.

## Supplementary Material

jeag225_Supplementary_Data

## Data Availability

The data underlying this article will be shared on reasonable request to the corresponding author.
