# Supplementary material for: Platelet activation and thrombosis in microvascular obstruction
Source: Eur Heart J Cardiovasc Imaging. 2026 Aug 30;27(11):1939–52. doi: 10.1093/ehjci/jeag225 (PMC13721768; doi:10.1093/ehjci/jeag225)
Supplement: Supplementary file 1 [file jeag225_supplementary_data.docx]

**Supplementary Material**

**Section 1: Reproducibility of Myocardial [¹⁸F]GP1 Quantification**

[¹⁸F]GP1 PET quantification was performed by two independent experienced readers blinded to clinical and cardiac MR data. Reproducibility was assessed using intraclass correlation coefficients (ICC) and Bland–Altman analysis for both FusionQuant and QPET software platforms.

**Supplementary Table 1. Intra-observer and inter-observer comparison of myocardial [¹⁸F]GP1 uptake measurements (FusionQuant).**

*Mean values ± standard deviation. P values derived from paired comparisons between measurements. No significant differences were observed across all metrics, supporting excellent reproducibility.*

|  | **Mean 1 ± SD** | **Mean 2 ± SD** | **P value** |
| --- | --- | --- | --- |
| **Myocardial intra-observer (FusionQuant)** | | | |
| SUVmean | 1.60±0.91 | 1.61±0.96 | 0.962 |
| SUVmax | 2.29±1.32 | 2.28±1.34 | 0.993 |
| TBRmean | 1.11±0.75 | 1.10±0.77 | 0.854 |
| TBRmax | 1.57±1.03 | 1.56±1.04 | 0.976 |
| **Myocardial inter-observer (FusionQuant)** | | | |
| SUVmean | 1.61±0.91 | 1.63±0.97 | 0.156 |
| SUVmax | 2.29±1.32 | 2.31±1.36 | 0.957 |
| TBRmean | 1.11±0.75 | 1.13±0.78 | 0.246 |
| TBRmax | 1.57±1.04 | 1.59±1.06 | 0.947 |

**Supplementary Table 2. Reproducibility of myocardial [¹⁸F]GP1 uptake using intraclass correlation coefficients and Bland–Altman analysis (FusionQuant).**

*ICC = intraclass correlation coefficient; CI = confidence interval; LOA = limits of agreement.*

|  | **ICC (95% CI)** | **Mean of Differences (Limits of Agreement)** |
| --- | --- | --- |
| **\Myocardial intra-observer (FusionQuant)** | | |
| SUVmean | 0.993 (0.983 to 0.997) | −0.010 (−0.218 to 0.216) |
| SUVmax | 0.996 (0.994 to 0.997) | 0.003 (−0.126 to 0.133) |
| TBRmean | 0.997 (0.995 to 0.999) | 0.002 (−0.095 to 0.099) |
| TBRmax | 0.998 (0.997 to 0.999) | 0.006 (−0.048 to 0.061) |
| **Myocardial inter-observer (FusionQuant)** | | |
| SUVmean | 0.990 (0.974 to 0.996) | −0.020 (−0.291 to 0.250) |
| SUVmax | 0.992 (0.898 to 0.994) | −0.023 (−0.220 to 0.175) |
| TBRmean | 0.996 (0.991 to 0.999) | −0.017 (−0.143 to 0.109) |
| TBRmax | 0.997 (0.992 to 0.999) | −0.022 (−0.183 to 0.139) |

**Supplementary Table 3. Intra-observer and inter-observer comparison of myocardial [¹⁸F]GP1 uptake measurements (QPET).**

*Mean values ± standard deviation. SD = standard deviation.*

|  | **Mean 1 ± SD** | **Mean 2 ± SD** | **P value** |
| --- | --- | --- | --- |
| **Myocardial intra-observer (QPET)** | | | |
| SUVmean | 1.22±0.35 | 1.24±0.34 | 0.713 |
| SUVmax | 1.66±0.49 | 1.64±0.54 | 0.598 |
| TBRmean | 0.73±0.20 | 0.75±0.23 | 0.578 |
| TBRmax | 1.39±0.70 | 1.41±0.74 | 0.324 |
| Percentage myocardial platelet activation (%) | 11.08±15.11 | 11.12±15.22 | 0.778 |
| **Myocardial inter-observer (QPET)** | | | |
| SUVmean | 1.23±0.33 | 1.24±0.35 | 0.711 |
| SUVmax | 1.65±0.51 | 1.67±0.56 | 0.628 |
| TBRmean | 0.74±0.22 | 0.75±0.21 | 0.574 |
| TBRmax | 1.42±0.71 | 1.45±0.85 | 0.319 |
| Percentage myocardial platelet activation (%) | 11.09±15.11 | 11.26±16.4 | 0.778 |

**Supplementary Table 4. Reproducibility of myocardial [¹⁸F]GP1 uptake using intraclass correlation coefficients and Bland–Altman analysis (QPET).**

*ICC = intraclass correlation coefficient; CI = confidence interval; LOA = limits of agreement. Although reproducibility metrics were marginally lower with QPET than FusionQuant for some parameters, agreement remained firmly within the good-to-excellent range.*

|  | **ICC (95% CI)** | **Mean of Differences (Limits of Agreement)** |
| --- | --- | --- |
| **Myocardial intra-observer (QPET)** | | |
| SUVmean | 0.940 (0.911 to 0.960) | −0.004 (−0.235 to 0.226) |
| SUVmax | 0.887 (0.835 to 0.924) | 0.013 (−0.465 to 0.491) |
| TBRmean | 0.961 (0.941 to 0.974) | −0.004 (−0.125 to 0.118) |
| TBRmax | 0.981 (0.972 to 0.988) | −0.014 (−0.288 to 0.259) |
| Percentage myocardial platelet activation (%) | 0.995 (0.993 to 0.997) | −0.044 (−3.02 to 2.932) |
| **Myocardial inter-observer (QPET)** | | |
| SUVmean | 0.915 (0.875 to 0.943) | −0.005 (−0.283 to 0.272) |
| SUVmax | 0.844 (0.774 to 0.893) | −0.015 (−0.593 to 0.563) |
| TBRmean | 0.931 (0.898 to 0.953) | −0.005 (−0.168 to 0.158) |
| TBRmax | 0.899 (0.852 to 0.932) | −0.036 (−0.720 to 0.648) |
| Percentage myocardial platelet activation (%) | 0.927 (0.892 to 0.950) | -0.176 (-12.078 to 11.726) |

**Section 2: Ischaemic Time Sensitivity Analyses**

To determine whether the main associations between myocardial [¹⁸F]GP1 uptake and microvascular obstruction, no-reflow and infarct size were influenced by late-presenting patients, formal interaction analyses and sensitivity analyses excluding patients with ischaemic times exceeding 24 hours (n=11) and 12 hours (n=20) were performed. No patients had ischaemic times exceeding 48 hours.

**Supplementary Table 5. Multivariable predictors of binary myocardial [¹⁸F]GP1 uptake: logistic regression across full cohort and ischaemic time-restricted sensitivity samples (odds ratio, 95% CI).**

| **Predictor** | **Full cohort (n=100)** | **Excl. >24h (n=89)** | **Excl. >12h (n=80)** |
| --- | --- | --- | --- |
| No-reflow | 16.15 (3.61 to 2.22), p<0.001 | 19.33 (3.77 to 99.25), p<0.001 | 19.69 (3.60 to 107.71), p<0.001 |
| Infarct burden (% late gadolinium enhancement) | 1.08 (0.99 to 1.18), p=0.084 | 1.04 (0.95 to 1.14), p=0.406 | 1.07 (0.96 to 1.19), p=0.206 |
| Degree of LGE | 2.75 (0.95 to 7.99), p=0.063 | 3.67 (1.18 to 11.41), p=0.025 | 2.51 (0.73 to 8.62), p=0.145 |
| Ejection fraction on cardiac MR (%) | 1.00 (0.92 to 1.09), p=0.986 | 0.99 (0.91 to 1.08), p=0.883 | 0.99 (0.90 to 1.08), p=0.773 |

**Supplementary Table 6. Independent predictors of microvascular obstruction burden (% LV): linear regression across full cohort and ischaemic time-restricted sensitivity samples (β coefficient, 95% CI).**

| **Predictor** | **Full (n=100, R²=0.70)** | **Excl. >24h (n=89, R²=0.72)** | **Excl. >12h (n=80, R²=0.72)** |
| --- | --- | --- | --- |
| Myocardial [¹⁸F]GP1 burden (%) | 0.140 (0.095 to 0.184), p<0.001 | 0.137 (0.094 to 0.179), p<0.001 | 0.144 (0.098 to 0.189), p<0.001 |
| No-reflow | 3.21 (1.87 to 4.56), p<0.001 | 3.20 (1.91 to 4.49), p<0.001 | 3.08 (1.75 to 4.42), p<0.001 |
| Ischaemic time (hours) | 0.039 (0.020 to 0.057), p<0.001 | 0.043 (−0.046 to 0.133), p=0.341 | −0.196 (−0.423 to 0.031), p=0.089 |
| Age (years) | −0.041 (−0.090 to 0.009), p=0.105 | −0.032 (−0.077 to 0.013), p=0.161 | −0.027 (−0.072 to 0.018), p=0.240 |
| Baseline TIMI flow grade | 0.186 (−0.208 to 0.579), p=0.351 | 0.165 (−0.193 to 0.522), p=0.363 | 0.172 (−0.187 to 0.530), p=0.343 |

**Supplementary Table 7. Independent predictors of infarct size (% LV): linear regression across full cohort and ischaemic time-restricted sensitivity samples (β coefficient, 95% CI).**

| **Predictor** | **Full (n=100, R²=0.55)** | **Excl. >24h (n=89, R²=0.53)** | **Excl. >12h (n=80, R²=0.52)** |
| --- | --- | --- | --- |
| Myocardial [¹⁸F]GP1 burden (%) | 0.462 (0.291 to 0.634), p<0.001 | 0.424 (0.240 to 0.608), p<0.001 | 0.491 (0.283 to 0.699), p<0.001 |
| No-reflow | 2.36 (−2.82 to 7.55), p=0.368 | 3.44 (−2.13 to 9.02), p=0.223 | 2.35 (−3.78 to 8.48), p=0.448 |
| Ischaemic time (hours)† | 0.122 (0.050 to 0.193), p=0.001 | −0.106 (−0.494 to 0.282), p=0.589 | −0.365 (−1.405 to 0.676), p=0.487 |
| Age (years) | −0.234 (−0.425 to −0.043), p=0.017 | −0.239 (−0.433 to −0.045), p=0.016 | −0.201 (−0.409 to 0.007), p=0.059 |
| Baseline TIMI flow grade | −1.534 (−3.051 to −0.018), p=0.047 | −1.955 (−3.503 to −0.406), p=0.014 | −1.638 (−3.282 to 0.006), p=0.051 |

**Section 3: Collinearity Assessment and Independent Predictors of Microvascular Obstruction and Infarct Size**

Variance inflation factors (VIF) were calculated for all covariates in the primary multivariable model (Table 2 of manuscript) and in two new models with percentage microvascular obstruction and infarct size as dependent variables. All VIF values were well below thresholds of concern (>5–10), confirming the absence of problematic collinearity. Sensitivity analyses excluding angiographic no-reflow are presented alongside primary models.

S**upplementary Table 8. Collinearity assessment: variance inflation factors for all covariates in the existing multivariable model predicting binary myocardial [¹⁸F]GP1 uptake (Table 2 of manuscript).**

| **Predictor** | **VIF** |
| --- | --- |
| No-reflow | 1.52 |
| Infarct burden (% late gadolinium enhancement) | 3.27 |
| Degree of LGE | 2.85 |
| Ejection fraction on cardiac MR (%) | 2.00 |

**Supplementary Table 9. Multivariable predictors of microvascular obstruction burden (% left ventricular mass): linear regression in the full cohort and sensitivity analysis excluding angiographic no-reflow, with variance inflation factors.**

| **Predictor** | **Full model (n=100, R²=0.70)** | **VIF** | **Sensitivity — excl. no-reflow (n=100, R²=0.63)** | **VIF** |
| --- | --- | --- | --- | --- |
| Myocardial [¹⁸F]GP1 burden (%) | 0.140 (0.095 to 0.184), p<0.001 | 1.88 | 0.203 (0.164 to 0.243), p<0.001 | 1.20 |
| Angiographic no-reflow | 3.210 (1.865 to 4.556), p<0.001 | 1.87 | — | — |
| Ischaemic time (hours) | 0.039 (0.020 to 0.057), p<0.001 | 1.15 | 0.051 (0.031 to 0.071), p<0.001 | 1.06 |
| Age (years) | −0.041 (−0.090 to 0.009), p=0.105 | 1.06 | −0.048 (−0.103 to 0.007), p=0.086 | 1.06 |
| Baseline TIMI flow grade | 0.186 (−0.208 to 0.579), p=0.351 | 1.26 | 0.115 (−0.319 to 0.549), p=0.600 | 1.25 |

**Supplementary Table 10. Multivariable predictors of infarct size (% left ventricular mass defined by late gadolinium enhancement): linear regression in the full cohort and sensitivity analysis excluding angiographic no-reflow, with variance inflation factors.**

| **Predictor** | **Full model (n=100, R²=0.55)** | **VIF** | **Sensitivity — excl. no-reflow (n=100, R²=0.55)** | **VIF** |
| --- | --- | --- | --- | --- |
| Myocardial [¹⁸F]GP1 burden (%) | 0.462 (0.291 to 0.634), p<0.001 | 1.88 | 0.509 (0.372 to 0.646), p<0.001 | 1.20 |
| Angiographic no-reflow | 2.363 (−2.822 to 7.548), p=0.368 | 1.87 | — | — |
| Ischaemic time (hours) | 0.122 (0.050 to 0.193), p=0.001 | 1.15 | 0.131 (0.062 to 0.199), p<0.001 | 1.06 |
| Age (years) | −0.234 (−0.425 to −0.043), p=0.017 | 1.06 | −0.239 (−0.429 to −0.048), p=0.015 | 1.06 |
| Baseline TIMI flow grade | −1.534 (−3.051 to −0.018), p=0.047 | 1.26 | −1.586 (−3.097 to −0.076), p=0.040 | 1.25 |

**Supplementary Table 11. Unique variance in microvascular obstruction and infarct size independently explained by myocardial [¹⁸F]GP1 burden versus angiographic no-reflow: semi-partial R² analysis and nested model comparison.**

*Unique R² for each predictor represents the reduction in model R² when that predictor is removed from the full model with all other covariates retained.*

|  | **Model A: MVO burden** | **Model B: Infarct size** |
| --- | --- | --- |
| Full model R² | 0.700 | 0.554 |
| Myocardial [¹⁸F]GP1 burden — unique R² | 0.123 (12.3%) | 0.136 (13.6%) |
| Angiographic no-reflow — unique R² | 0.071 (7.1%) | 0.004 (0.4%) |
| Nested F-test: removing no-reflow | F=22.45, p<0.001 | F=0.82, p=0.37 |
